# Factors associated with self-reported health status among older adults in the Greater Kampala Metropolitan Area, Uganda: a cross-sectional survey

**DOI:** 10.1080/16549716.2026.2748751

**Published:** 2026-11-02

**Authors:** Jude Otim, Valérie Golaz, Stephen Ojiambo Wandera, Charles Lwanga, Pierre Schlegel, John Bosco Asiimwe, Patricia Ndugga, Betty Kwagala

**Affiliations:** a School of Statistics and Planning, Makerere University, Kampala, Uganda; b Institut national d’études démographiques (INED), Paris, France; c Aix-Marseille University, IRD, LPED, Marseille, France

**Keywords:** Subjective health, ageing, sex, older people, urban Uganda

## Abstract

**Background:**

Literature on the prevalence and factors associated with good self-reported health among older adults in the Greater Kampala Metropolitan Area (GKMA), including sex-stratified analyses, remains limited.

**Objectives:**

This study aimed to examine the prevalence of good self-reported health and its associated factors among older adults (50–64 years) in the GKMA, with sex-stratified analyses.

**Methods:**

Data stem from the ‘The Remaking of the Family in East Africa (FAMILEA)’ survey of 2024. A set of cross-sectional variables from a subsample of 651 older adults (aged 50–64) was used. We applied frequency distributions for descriptive statistics and a multivariable modified Poisson regression model to investigate factors associated with self-reported health status among older adults in the GKMA, with sex-stratified patterns.

**Results:**

In the GKMA, 58% of older adults (64% of males and 52% of females) declared good health. Older adults living in Wakiso District, satisfied with accommodation, working, not living in a household-owned house, in the second and fourth quintiles, and not functionally constrained, reported good health. Accommodation satisfaction and non-limited functional status were positively associated with good health, irrespective of sex. Among males, living in a nuclear family, working, not reporting the lowest wealth quintile, and being affiliated with the Catholic religion were positively associated with good health. Among females, living in Wakiso District and never lacking companionship were positively associated with good health.

**Conclusion:**

Good self-reported health was associated with various factors among older adults (50–64 years) in the GKMA, with some patterns observed in the sex-stratified models.

## Paper Context

- **Main findings:** Among older adults, good health was associated with district of residence, satisfaction with accommodation, working status, house ownership, wealth quintile, and functional status. Sex-stratified analyses showed potential differences in factors associated with good self-reported health among older adults aged 50–64 in the Greater Kampala Metropolitan Area. Sex-stratified findings should be cautiously interpreted as suggestive and exploratory rather than definitive because the estimates may be unstable due to limited statistical power. In the sex-stratified descriptive analyses, a higher share of males reported good health. Accommodation satisfaction and non-functional limitation status showed a positive significant association with good self-reported health in the full model, with similar patterns observed in the sex-stratified models.
- **Added knowledge:** This study expands knowledge of this rapidly developing topic in two major ways: First, by investigating the prevalence of good self-reported health among older adults in the Greater Kampala Metropolitan Area, alongside sex stratification. Second, by examining factors associated with good self-reported health among older adults in the Greater Kampala Metropolitan Area, along with sex-stratified pattern analyses.
- **Global health impact for policy and action:** This research highlights the need to advance health equity in the urban populations, particularly for the growing population of older adults in the metropolitan area. The study also underscores the possibility of sex-specific disparities in factors associated with good self-reported health among older adults in the metropolitan area. This suggests that future research should explore whether design and improvement approaches need to consider these patterns once they are well established. Thus, incorporating demographic, socioeconomic, and health-related correlates into the country’s ageing policy agenda is especially crucial for delivering context-specific public health interventions.

## Background

As the global population ages, older adults’ health status has become central to public health planning, policy, and research. During recent decades, the population of people aged 60 and older in both developed and developing worlds has increased. According to the World Health Organization (WHO), the population of older persons is projected to increase from 1.1 billion in 2023 to 1.4 billion in 2030, and to 2.1 billion by 2050 [1,2]. In Uganda, available statistics show that the number of older persons aged 60 and older rose from 1.3 million in 2010 to 2 million by 2024 [3,4]; and by 2050, the number is projected to be over 6 million [5]. Although some studies report older people to be aged 60 and older [6,7], numerous investigations in low- and middle-income countries use a threshold of 50 years and older [8–14], attributed to the low life expectancy [15]. Therefore, though there is an undeniable rise in the number of older persons, the increase presents unique and complex challenges to the Ugandan healthcare sector, which is often underfunded, struggling with a high burden of infectious diseases and reproductive-related health issues. However, to achieve good health and well-being for all by 2030 (Sustainable Development Goal 3), equity and equality in healthcare for older adults are indispensable.

On the one hand, the objective of health-related research and diagnosis in the developing world is to provide essential data. Due to limited health infrastructure, these efforts fail to capture the full spectrum of older adults’ health experiences. This significant data gap emphasises the need to integrate self-reported health status (SRH) as complementary information in assessing the mental, physical, and social well-being of people, including older adults [16,17]. Though Self-reported health status is subjective, it serves as a powerful indicator of healthcare utilisation and mortality [18–20]. For instance, Zadworna argued that self-reported health among older adults is linked to healthy ageing [21]. In Uganda, previous studies have associated the vulnerability of older adults with poor SRH status [5,10]. Indeed, in countries with resource constraints, the use of SRH is cost-effective, gives older adults a voice to communicate their health concerns, and allows policymakers to identify health challenges, allocate resources, and design culturally appropriate interventions [16,22].

Accompanying the need to integrate SRH when exploring the variations in reporting health status, it is important to analyse the past experiences of older adults along with their individual, cultural, economic, historical, and institutional factors [23]. Other studies have also reported sex variations in the self-assessment of health [24]. Using 2007 data from an investigation on Global Ageing and Adult Health (SAGE) wave 1 in Ghana, Fonta et al. attributed this to the lack of socioeconomic factors, resulting in the incapacity to respond to health needs [25]. Evidence from previous studies show that older females report better health [26,27]. However, several studies have found that older males have better self-reported health [10,28].

Several socioeconomic, demographic, and health-related factors, including social participation, smoking, alcohol intake, education level, wealth quintile, employment, religious affiliation, house ownership, age, place of residence, sex, marital status, family structure, living arrangements, household headship, and functional status, were related to self-reported ill health [10,26,29–31]. In addition, there is a relationship between sociocultural factors and people’s self-reported health. Golaz et al., for instance, argued that the weakening of the family support system is associated with older adults’ health [32]. When family support systems decline, older adults become increasingly vulnerable to loneliness, financial instability, and a lack of care. Evidence also shows that being widowed or divorced is associated with poor health [33].

## Rationale

Numerous studies in Uganda have examined the association between older adults’ health status and sociodemographic factors, including access to and use of healthcare services [34]; correlates of self-reported ill health status [10]; and the prevalence and associated factors of living alone among older adults in Uganda [11,35]. The aforementioned studies did not examine self-reported health status in a metropolitan setting, along with a sex dimension. Metropolitan areas present a complex environment that can influence older adults’ health. For instance, a study by Adams-Price et al. among older adults aged 55 and older in Mississippi revealed that metropolitan areas provide a conducive environment for healthy ageing [36]. On the contrary, a study in the Maputo metropolitan area revealed that older adults aged 60 and older were more likely to report poor health [37].

The Greater Kampala Metropolitan Area, comprising Kampala, Wakiso, and Mukono, is characterised by the largest population in the country of about 6.1 million (13%) in 2024, up from 4.1 million (12%) in 2014, including older adults [38,39], with limited literature on the prevalence of self-reported health status and its associated factors among older adults in the GKMA, as well as sex-stratified differences. Assessment of older adults’ health in the metropolitan area is essential, given the complex interaction of demographic, socioeconomic, and health-related factors, as well as changing cultural conditions, that affect their well-being. The current study utilised data on self-reported health status to examine its prevalence and associated factors among older adults in a metropolitan setting and to assess sex-stratified patterns. Understanding what explains SRH in the Greater Kampala Metropolitan Area (GKMA) is expected to identify correlates, highlight sex-based patterns, gain insight into older adults’ unmet needs, and contribute to evidence-based policy formation and programming. Thus, the study aims to approximate the prevalence of good self-reported health and the factors associated with it among older adults in the GKMA. It also aims to examine the sex-stratified patterns.

## Methods

### Study design, data setting

This study used data from a cross-sectional survey conducted in the Greater Kampala Metropolitan Area, Uganda. The FAMILEA-Greater Kampala Metropolitan Area survey was part of a larger survey conducted in both Uganda and Kenya. With permission from the ‘*The Remaking of the Family in East Africa (FAMILEA)*’ project, data for this study were extracted from the project database.

The main objective was to map the shifting of family ideals, practices, and configurations in East Africa. The FAMILEA-Greater Kampala Metropolitan Area survey was implemented by the African Population and Health Research Centre (APHRC) and the Makerere University Department of Population Studies. Data were collected between 15 July and 11 August 2024.

### Study participants and sample size

The FAMILEA-Greater Kampala Metropolitan Area database presents data from a representative sample of 2000 respondents (males and females, aged 18–64), at the individual level on individual family situations and well-being, household structure, relationships with extended family members, and social networks, among other themes. This survey was conducted in accordance with the Declaration of Helsinki.

In the implementation stage, a two-stage sampling procedure was employed. First, they sought technical assistance from the Uganda Bureau of Statistics (UBOS) to randomly select 100 Enumeration Areas (EAs) representative of the Greater Kampala Metropolitan Area (comprising Kampala, Wakiso, and Mukono Districts). Second, 20 individuals stratified by sex and age group (18–34, 35–49, 50–64) were selected using systematic sampling based on a random walk and a Kish grid from eligible regular household members.

The subsample for the current study comprises people aged 50–64, yielding a sample of 651 individuals, excluding 2 individuals who did not know or refused to answer the question ‘How is your health in general?’ We applied a threshold of 50 based on studies using the Indepth Network and World Health Organization data, defining older adults beginning at the age of 50 years [8–12,14,40–42]. The 50–64 age range was adopted because of its relationship with a significant increase in chronic conditions and restricted ability in certain mobility-related tasks, reduced engagement in activities that entail the generation of income, and an increase in the financial burden of health disturbances [43–45]. Thus, comprehension of how these older adults assess their health supports early detection of discrepancies, informs preventive strategies, and guides policies aimed at promoting healthy ageing. Figure 1 is a flow diagram that shows the origin of the study sample.

**Figure 1.**
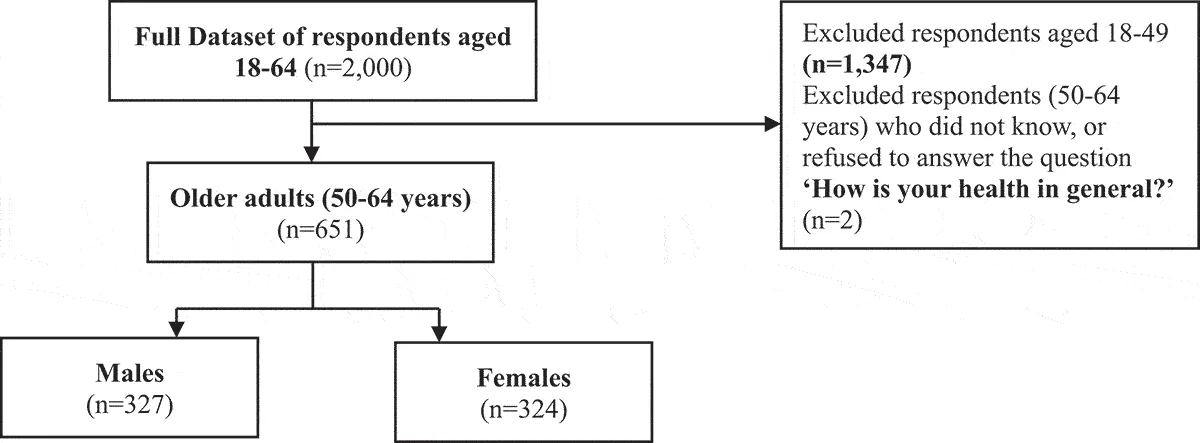
Origin of the sample used in the study.

### Variables and measures

During data collection, the FAMILEA study measured self-reported health status using the general question, ‘How is your health in general?’ with responses captured as ‘very good, good, fair, bad, and very bad. The outcome variable was created as a binary variable from the above-mentioned responses, indicating whether the health status of the older adults is good (good self-reported health, coded as “1”) or poor (poor self-reported health, coded as “0”)’. Good self-reported health included responses ‘very good’ and ‘good’, and poor self-reported health included responses ‘fair’, ‘bad’, and ‘very bad’. This dichotomous classification of self-reported health status has been supported in several studies [46–49]. Do not know, and refusals were excluded from the analysis to ensure analytical precision, given the small number of such responses (*n* = 2).

Independent variables were categorised as demographic, socioeconomic, and health-related variables. Demographic variables included sex, age, marital/union status, family structure, and district of residence. During data collection, sex was recorded as a binary variable (male and female) and used as recorded during data analysis. In this study, age is a filter variable, as it identifies the age group under study. It was recorded as a continuous variable, and during analysis, it was grouped into three categories: 50–54, 55–59, and 60–64. The 5-year age grouping is a standard demographic grouping [50,51]. Marital/union status was captured as yes and no, indicating whether the respondent has a spouse/partner or not, and was modelled as ‘yes or no. The family structure was constructed from household composition and corresponding relationships and recorded as nuclear family structure, extended family structure, and alternative family structure. Alternative family structures were grouped to include respondents who reported living alone, single parenting, couple alone, with non-kin, non-kin and children, and couple and non-kin. Alternative family structures comprise families that deviate from the traditional nuclear and extended family models, such as living alone, single-parent, childless, stepparent, kinless, and other non-families, which entail a wide variety of non-traditional configurations [52–55]. These are structures typically founded on choice instead of blood and marital ties [56,57]. We merged all alternative categories for statistical stability and validity [58]; the district of residence was captured and used as Wakiso, Mukono, and Kampala.

Socioeconomic variables included education level, working status, house ownership, religious affiliation, wealth quintile, lack of companionship, frequency of contact with kin, and accommodation satisfaction. During data collection, Education level was computed and grouped as no education, primary, secondary, and tertiary from two questions: ‘What is the highest level of school you attended? and have you ever attended school? In modelling using the modified Poisson regression model, the variable was collapsed into three categories: ‘no education and primary, secondary, and tertiary. A combination of ‘no education and primary’ is epidemiologically justified in the context of developing countries, where differences between older adults with no or primary education have negligible variations in healthcare utilisation [59]. Based on several indicators, having primary education has negligible variation with having no education, because the benefits of education are only significant with secondary education [60]. The variable working status was grouped as working and not working; house ownership was categorised as owner and not owner. Religious affiliation was captured and used as Catholic, Anglican, Pentecostal, Muslim, and other. Muslims were combined with others, including no religion, Orthodox, Seventh-day Adventists, and Jehovah’s Witnesses, because there were few cases of these affiliations.

To construct wealth quintiles from assets and household characteristics, we used the Demographic and Health Survey (DHS) approach based on Principal Component Analysis (PCA) to construct wealth quintiles [61,62]. We also applied Multiple Correspondence Analysis (MCA) [63] in a separate stage to examine associations among categorical variables and assess robustness. MCA was applied as a diagnostic criterion, and it did not change the variables selected for PCA. Both MCA and PCA were used independently to assess robustness (high agreement) and sensitivity (poor agreement) of quintile categorisation [64]. Five groups of wealth quintiles in four dimensions were generated. This study identified wealth quintiles as the lowest, second, middle, fourth, and highest. Lack of companionship was captured as a continuous variable indicating the number of times in the past 4 weeks a respondent felt lack of companionship and in analysis, it was categorized as never, rarely, sometimes and often (3+ times a week); similarly, the number of times a respondent is in contact with kin using either electronic/phone, post or physical meetings was used to collect information on the variable ‘frequency of contact with kin’. Responses for this variable were constructed from the frequency of contacts with twelve (12) categories of kin members, including spouse, children, biological mother and father, guardians, parents-in-law, biological brothers and sisters, step-siblings, cousins, and family members on both maternal and paternal sides. It is from these that data was captured using a list of nine responses: ‘daily/almost daily, weekly, monthly, twice a year, once a year, very rarely or never, don’t know, refusal and not applicable’. However, during analysis, this was reduced to four groups as ‘at least once a week or month (for daily/almost daily, weekly, monthly), twice a year, once a year, and very rarely or never’. Do not know, refuse, and not applicable were not grouped because they were non-committal.

During data collection, respondents were asked how satisfied they were with their accommodation. Respondents’ responses ranged from not satisfied at all to very satisfied, and in the analysis, this variable was presented as not satisfied (not satisfied at all, not satisfied, and fair) and satisfied (satisfied and very satisfied). One’s health-related problems shape one’s functional status. During data collection, the functional status variable was captured for the 6 months prior to the survey as severely limited, limited but not severely, not limited, refusal, and I do not know. In the modelling, three cases constituting 0.46% were excluded because they were non-committal. One respondent refused to answer, and two said that they did not know. ‘Severely limited’ and ‘limited but not severely’ were merged to form a code, denoted as ‘limited’. Thus, a dummy variable for functional status was collapsed into two groups: not limited and limited.

This study utilised a secondary dataset without a priori sample size estimation. However, we employed survey weights and adjusted for clustering to ensure representativeness in estimates [65,66].

### Bias assessment

This study uses data that may be subject to social desirability bias. Specifically, on age reporting and household asset ownership.

### Statistical methods

We applied sampling weights to correct for imbalances in selection probabilities and non-response bias and used the *svy* prefix (Stata 18) to account for the survey’s stratified, clustered design, thereby producing population-representative estimates with standard errors consistent with the survey design. Descriptive statistics stratified by sex were presented as frequencies and percentages to describe the study population. Chi-square tests were performed, with a significance level of *p* < 0.05. These were used in the analysis to establish the relationship between the outcome and the independent variable. In the data, approximately 58% reported good health status, indicating that this was a common event. To isolate the net independent association of each independent variable on the self-reported health status of older adults, we applied a modified Poisson regression model [67].

The use of this model is based on three arguments: First, when the outcome variable is common, this model does not overestimate the risk ratio (RR), as it would if other binary models, such as logistic or probit regression, were used. Second, it directly estimates the risk ratio. Third, it does not suffer from computational problems when it comes to convergence while fitting the model [67–73]. We reported a significant threshold of 0.05 and an Adjusted Prevalence Ratio (aPR) with a 95% Confidence Interval (CI).

Before fitting the model, the independent variables were tested for collinearity using the variance inflation factor (VIF). Highly correlated variables with a VIF of over 10 (indicating severe multicollinearity) were dropped, and the diagnostics showed a mean VIF of 1.53. The fitted model was subjected to a *link test* to assess whether the independent variables were appropriately fitted [74,75]. The link test uses *hat* and *_hat-squared* statistics. Because *_hat* is the predicted value from the model, it should be significant (*p* < 0.05), and when the model describes the data correctly, the *_hat-squared*, which represents misspecification, should not be significant (*_hat-squared*, p*>*0.05), which implies that the observed data mirrors the expected. In this investigation, we assumed that the missing data (16 cases) were missing completely at random (MCAR), and inferences were not biased [76,77]. We examined missing data using two approaches. First, the global MCAR test established that the study respondents with complete versus missing data on respondents’ marital status/union status, lack of companionship, frequency of contact with kin, functional status, and education level were not statistically different (*p* > 0.05) across age (Table 1). Second, we then executed Little’s MCAR test to evaluate the global MCAR test, whose result was not statistically significant (χ^2^ = 31.09, df = 26, *p* = 0.225). Both tests support the assumption that the data are Missing Completely at Random (MCAR).

**Table 1.** Distribution of missingness in marital/union status, lack of companionship, frequency of contact with kin, education level, and functional status.

Variable (Missingness)	Age group	n	Missing (%)	p-value ($\mathrm{ch}i^{2})$
Marital/union status				**0.57 (χ**^**2**^** = 1.12)**
	50–54	320	2 (0.65)	
	55–59	155	1 (0.65)	
	60–64	176	0 (0.00)	
Lack of companionship				**0.18 (χ**^**2**^** = 3.38)**
	50–54	320	3 (0.94)	
	55–59	155	3 (1.94)	
	60–64	176	0 (0.00)	
Frequency of contact with kin				**0.44 (χ**^**2**^** = 1.64)**
	50–54	320	3 (0.94)	
	55–59	155	1 (0.65)	
	60–64	176	0 (0.00)	
Education level				**0.35 (χ**^**2**^** = 2.08)**
	50–54	320	2 (0.62)	
	55–59	155	0 (0.00)	
	60–64	176	0 (0.00)	
Functional status				**0.19 (χ**^**2**^** = 3.29)**
	50–54	320	1 (0.31)	
	55–59	155	2 (1.29)	
	60–64	176	0 (0.00)	

## Results

### Distribution of respondents by demographic, socioeconomic, and health-related characteristics

Table 2 presents the distribution of respondents (50–64 years) by demographic, socioeconomic and health-related characteristics in the GKMA, alongside sex stratification. Results showed that females were slightly more represented than males (53%), and half of the older adults were aged 50–54. The average age of respondents was about 56 years, with a standard deviation of 4.6 (not in table). Approximately 64% had spouses or partners, and approximately 49% lived with extended family members. Among all the older adults included in the analysis, 48% were from Wakiso District, 41% from Kampala District, and 11% from Mukono District. Regarding lack of companionship, approximately 42% had never felt a lack of companionship in the past 4 months, but the majority (58%) had felt a lack of companionship. The majority of older adults (71%) were satisfied with their accommodation, 52% had at most primary-level education, 75% were working or employed, and 72% were living in a house owned by the household. Concerning religious affiliation, 34% were Catholic, 30% Protestant, 14% Pentecostal, and 22% Muslim or others. Approximately 13%, 15%, 20%, 23%, and 28% were in the lowest, second, middle, fourth, and highest quintiles, respectively. About 62% of the participants had no functional limitations.

**Table 2:** Distribution of demographic, socioeconomic, and health-related characteristics of older adults in the Greater Kampala Metropolitan Area, alongside sex stratification.

Variables	Sex						
	Males		Females		All		
	(n=327)		(n=324)		(n=651)		
	Valid N	n (%)	Valid N	n (%)	Valid N	n (%)	p-value
**Sex**					651		
Male						327 (47.1)	
Female						324 (52.9)	
**Age group**	327		324		651		0.90
50 – 54 years		160 (49.4)		160 (49.8)		320 (49.6)	
55 – 59 years		75 (23.3)		80 (24.5)		155 (24.0)	
60 – 64 years		92 (27.3)		84 (25.6)		176 (26.4)	
**Marital/union status**	326		322		648		**<0.01**
No spouse/partner		55 (11.6)		206 (58.0)		261 (36.1)	
With spouse/partner		271 (88.4)		116 (42.0)		387 (63.9)	
**Family structure**	327		324		651		**<0.01**
Nuclear		127 (44.4)		43 (15.3)		170 (29.0)	
Extended		104 (39.7)		175 (57.8)		279 (49.3)	
Alternative		96 (15.9)		106 (26.9)		202 (21.7)	
**District of residence**	327		324		651		0.85
Wakiso		154 (46.2)		152 (48.6)		306 (47.5)	
Mukono		48 (11.7)		45 (11.1)		93 (11.4)	
Kampala		125 (42.1)		127 (40.3)		252 (41.1)	
**Education level**	327		322		649		**<0.01**
No education/Primary		147 (43.7)		202 (59.8)		349 (52.2)	
Secondary		113 (33.3)		91 (29.8)		204 (31.5)	
Tertiary		67 (23.0)		29 (10.4)		96 (16.3)	
**Working status**	327		324		651		**<0.01**
Not working		65 (18.9)		96 (30.3)		161 (25.0)	
Working		262 (81.1)		228 (69.7)		490 (75.0)	
**House ownership** 327			324		651		0.98
Owner		216 (72.2)		229 (72.2)		445 (72.2)	
Not owner		111 (27.8)		95 (27.8)		206 (27.8)	
**Religious affiliation**	327		324		651		0.56
Catholic		107 (35.3)		107 (33.0)		214 (34.1)	
Protestant		102 (27.2)		96 (32.6)		198 (30.1)	
Pentecostal		42 (14.0)		50 (14.2)		92 (14.1)	
Muslim & others		76 (13.5)		71 (20.2)		147 (21.7)	
**Wealth quintile**	327		324		651		0.16
Lowest		43 (10.3)		53 (16.0)		96 (13.3)	
Second		51 (13.5)		55 (16.1)		106 (14.9)	
Middle		66 (20.8)		71 (20.0)		137 (20.4)	
Fourth		81 (23.0)		81 (23.5)		162 (23.3)	
Highest		86 (32.3)		64 (24.4)		150 (28.1)	
**Lack of companionship**^a^	324		321		645		**<0.01**
Never		174 (56.9)		97 (28.6)		271 (42.0)	
Rarely		50 (17.9)		99 (32.4)		149 (25.5)	
Sometimes		51 (13.1)		82 (25.2)		133 (19.5)	
Often (3+ times a week)		49 (12.1)		43 (13.8)		92 (13.0)	
**Frequency of contact with kin**^b^	326		321		647		**<0.01**
At least weekly/Monthly		147 (46.5)		62 (20.7)		209 (32.9)	
Twice a year		54 (16.5)		52 (15.0)		106 (15.7)	
Once a year		19 (5.5)		31 (9.9)		50 (7.8)	
Very rarely		106 (31.6)		176 (54.4)		282 (43.6)	
**Accommodation satisfaction** 327			324		651		**0.02**
Not satisfied		113 (34.2)		80 (24.5)		193 (29.1)	
Satisfied		214 (65.8)		244 (75.5)		458 (70.9)	
**Self-reported health status**	327		324		651		**<0.01**
Poor SRH		123 (35.9)		162 (48.4)		285 (42.5)	
Good SRH		204 (64.1)		162 (51.6)		366 (57.5)	
**Functional status**	326		322		648		**<0.01**
Limited		99 (29.7)		149 (45.7)		248 (38.1)	
Not limited		227 (70.3)		173 (54.3)		400 (61.9)	

n frequency, N total number, survey weights were applied ^a^Lack of companionship in the past four (4) months. ^b^Contact through the phone, electronic ways, post, and physical meetings.

Sex variations were observed: nearly 44% of males had at most primary-level education, compared with 60% of females. A higher proportion of older men (88%) than older women (42%) had a spouse/partner; 57% of older men never lacked companionship compared to 29% of older women, and 47% of older men contacted their kin at least weekly or monthly. In contrast, 46% of older females and 30% of older males had functional limitations.

### Differentials in self-reported health status among older adults (50–64 years) by demographic, socioeconomic, and health-related factors in the GKMA, alongside sex-stratification

Table 3 presents differentials in self-reported health status (SRH) among older adults by selected demographic, socioeconomic, and health-related characteristics in the GKMA, alongside sex stratification. Overall, SRH status varied significantly by marital/union status (χ^2^ = 9.89, *p* < 0.01), family structure (χ^2^ = 9.43, *p* = 0.02), district of residence (χ^2^ = 10.75, *p* < 0.01), lack of companionship (χ^2^ = 37.48, *p* < 0.01), accommodation satisfaction (χ^2^ = 44.32, *p* < 0.01), education level (χ^2^ = 10.36, *p* = 0.02), working status (χ^2^ = 15.89, *p* < 0.01), wealth quintile (χ^2^ = 25.01, *p* < 0.01), and functional status (χ^2^ = 183.97, *p* < 0.01). Table 3 also shows results stratified by sex. Among males, significant variation was observed by education level (χ^2^ = 21.76, *p* = 0.02); working status (χ^2^ = 30.94, *p* < 0.01); lack of companionship (χ^2^ = 24.32, *p* = 0.03); accommodation satisfaction (χ^2^ = 51.48, *p* < 0.01); and functional status (χ^2^ = 129.93, *p* < 0.01). Among females, SRH status varied by district of residence (χ^2^ = 18.76, *p* = 0.02); wealth quintile (χ^2^ = 38.49, *p* < 0.01); lack of companionship (χ^2^ = 45.66, *p* < 0.01); frequency of contact with kin (χ^2^ = 22.34, *p* = 0.03); accommodation satisfaction (χ^2^ = 49.71, *p* < 0.01); and functional status (χ^2^ = 220.59, *p* < 0.01). All variables were assessed at the bivariate stage, and those with theoretical relevance were maintained at the multivariable level, irrespective of their statistical significance in explaining the outcome [26,28,46,78–81]. This was performed to account for possible confounding variables [82].

**Table 3.** Differentials in self-reported health status among older adults (50–64 years) by demographic, socioeconomic, and health-related factors, and by sex in GKMA.

Variables	Sex								
	Males			Females			All		
	% Good SRH	Frequency (n)	p-value	% Good SRH	Frequency (n)	p-value	% Good SRH	Frequency (n)	p-value
**Sex**									**<0.01**
Male							64.1	204	
Female							51.6	162	
**Age group**			0.48			0.20			0.18
50 – 54 years	67.6	106		56.2	88		61.5	194	
55 – 59 years	63.0	48		42.0	32		51.6	80	
60 – 64 years	58.6	50		52.0	42		55.2	92	
**Marital/union status**			0.21			0.28			**<0.01**
No spouse/partner	54.0	27		48.4	100		49.2	127	
With spouse/partner	65.4	176		55.5	60		61.9	236	
**Family structure**			0.17			0.27			**0.02**
Nuclear	66.9	85		51.1	22		62.5	107	
Extended	65.9	70		55.4	94		59.4	164	
Alternative	51.6	49		43.7	46		46.5	95	
**District of residence**			0.30			**0.02**			**<0.01**
Wakiso	68.9	100		60.1	86		64.2	186	
Mukono	57.0	27		48.5	20		52.6	47	
Kampala	60.7	77		42.2	56		51.1	133	
**Education level**			**0.02**			0.67			**0.02**
No education/Primary	54.2	75		49.9	97		51.6	172	
Secondary	73.2	81		52.1	47		62.6	128	
Tertiary	69.6	48		59.8	17		66.3	65	
**Working status**			**<0.01**			0.18			**<0.01**
Not working	42.4	25		45.0	41		44.1	66	
Working	69.1	179		54.5	121		61.9	300	
**House ownership**			0.96			0.42			0.52
Owner	64.2	135		53.2	115		58.3	250	
Not owner	63.8	69		47.6	47		55.2	116	
**Religious affiliation**			0.35			0.28			0.45
Catholic	70.9	72		46.8	47		58.5	119	
Protestant	59.2	61		56.4	50		57.6	111	
Pentecostal	66.2	28		61.4	28		63.6	56	
Muslim & others	58.1	43		45.0	37		51.7	80	
**Wealth quintile**			0.10			**<0.01**			**<0.01**
Lowest	44.0	18		36.4	20		39.1	38	
Second	65.9	34		39.6	21		50.8	55	
Middle	66.5	40		43.8	35		54.7	75	
Fourth	58.0	46		62.4	47		60.4	93	
Highest	72.4	66		65.6	39		69.3	105	
**Lack of companionship**^a^			**0.03**			**<0.01**			**<0.01**
Never	69.1	120		64.4	57		67.4	177	
Rarely	68.2	34		61.2	58		63.5	92	
Sometimes	58.3	30		35.4	31		42.7	61	
Often (3+ times a week)	41.0	19		36.1	16		38.2	35	
**Frequency of contact with kin**			0.15			**0.03**			0.13
At least weekly/Monthly^b^	71.0	103		41.9	27		61.3	130	
Twice a year	62.5	29		42.1	23		52.2	52	
Once a year	66.0	14		73.9	22		71.3	36	
Very rarely	54.7	58		54.2	89		54.4	147	
**Accommodation satisfaction**			**<0.01**			**<0.01**			**<0.01**
Not satisfied	45.4	53		27.4	23		37.3	76	
Satisfied	73.8	151		59.5	139		65.7	290	
**Functional status**			**<0.01**			**<0.01**			**<0.01**
Limited	31.1	26		19.5	30		23.8	56	
Not limited	78.1	178		78.0	130		78.0	308	

Weighted proportions, *All* without selecting a particular type of sex, *SRH* Self-Reported Health. ^a^Lack of companionship in the past four (4) months. ^b^At least weekly were only 7 cases for the female.

### Multivariable results

Table 4 presents the three sets of nested models (for males, females, and both) constructed using the modified Poisson regression model. These included Model I, Model II, and the full model. Model I modelled the association between demographic factors and SRH status; in Model II, we adjusted for socioeconomic factors; and in the full model, we added functional status. Overall, good SRH was associated with district of residence, accommodation satisfaction, working status, house ownership, wealth quintile, and functional status. In Model I, where males and females were analysed together, females compared with males and older adults in Kampala district compared with Wakiso showed evidence of a 17% (aPR = 0.83) and 20% (aPR = 0.80) lower likelihood of reporting good SRH, respectively. Relatedly, females in Kampala district were 27% less likely to report good SRH (aPR = 0.73), and no variable among males showed evidence.

**Table 4.** Adjusted prevalence ratio (aPR) for self-reported health status among older adults in the GKMA, alongside sex-stratified patterns.

Variables	Sex											
	Male				Female				All			
	aPR	aPR	aPR	95% CI	aPR	aPR	aPR	95% CI	aPR	aPR	aPR	95% CI
	Model I	Model II	Full Model		Model I	Model II	Full Model		Model I	Model II	Full Model	
**Sex**												
Male**^rc^**									1.00	1.00	1.00	.
Female									0.83*	0.81*	0.93	0.80-1.10
**Age group**												
50 – 54 years**^rc^**	1.00	1.00	1.00	.	1.00	1.00	1.00	.	1.00	1.00	1.00	.
55 – 59 years	0.94	0.92	0.99	0.80-1.24	0.74	0.85	0.96	0.75-1.24	0.84	0.87	0.97	0.82-1.14
60 – 64 years	0.86	0.88	0.98	0.82-1.17	0.94	0.94	1.13	0.89-1.44	0.89	0.92	1.07	0.92-1.24
**Marital/union status**												
No spouse/partner**^rc^**	1.00	1.00	1.00	.	1.00	1.00	1.00	.	1.00	1.00	1.00	.
With spouse/partner	1.08	0.78	0.95	0.72-1.25	1.12	0.93	0.96	0.76-1.21	1.07	0.90	0.96	0.80-1.14
**Family structure**												
Nuclear**^rc^**	1.00	1.00	1.00	.	1.00	1.00	1.00	.	1.00	1.00	1.00	.
Extended	1.00	0.94	0.94	0.77-1.13	1.16	1.10	1.02	0.75-1.39	1.04	0.99	0.98	0.84-1.14
Alternative	0.78	0.72*	0.74*	0.57-0.94	0.99	0.93	1.02	0.70-1.49	0.84	0.82	0.87	0.72-1.07
**District of residence**												
Wakiso**^rc^**	1.00	1.00	1.00	.	1.00	1.00	1.00	.	1.00	1.00	1.00	.
Mukono	0.83	0.82	0.88	0.66-1.17	0.82	0.83	0.74	0.55-1.02	0.82	0.81	0.83	0.68-1.01
Kampala	0.87	0.85	0.90	0.76-1.06	0.73*	0.85	0.77*	0.60-0.98	0.80*	0.83*	0.83*	0.72-0.97
**Accommodation satisfaction**												
Not satisfied**^rc^**		1.00	1.00	.		1.00	1.00	.		1.00	1.00	.
Satisfied		1.58***	1.59***	1.25-2.03		1.83**	1.53*	1.06-2.21		1.66***	1.57***	1.27-1.93
**Education level**												
No education/Primary**^rc^**		1.00	1.00	.		1.00	1.00	.		1.00	1.00	.
Secondary		1.26*	1.19	0.98-1.45		0.95	0.92	0.72-1.16		1.08	1.04	0.90-1.20
Tertiary		1.11	1.10	0.86-1.42		0.97	0.99	0.76-1.32		1.03	1.02	0.85-1.22
**Working status**												
Not working**^rc^**		1.00	1.00	.		1.00	1.00	.		1.00	1.00	.
Working		1.55*	1.41*	1.03-1.93		1.27	1.11	0.89-1.39		1.33**	1.20*	1.00-1.44
**House ownership**												
Owner**^rc^**		1.00	1.00	.		1.00	1.00	.		1.00	1.00	.
Not owner		1.20	1.19	0.99-1.43		1.20	1.15	0.86-1.54		1.21*	1.18*	1.00-1.39
**Religious affiliation**												
Catholic**^rc^**		1.00	1.00	.		1.00	1.00	.		1.00	1.00	.
Protestant		0.80*	0.78*	0.63-0.96		1.18	1.13	0.88-1.45		0.97	0.95	0.82-1.10
Pentecostal		0.94	0.88	0.67-1.15		1.15	1.05	0.77-1.42		1.03	0.97	0.80-1.19
Muslim & others		0.90	0.87	0.71-1.07		0.96	0.99	0.73-1.36		0.93	0.90	0.75-1.07
**Wealth quintile**												
Lowest**^rc^**		1.00	1.00	.		1.00	1.00	.		1.00	1.00	.
Second		1.62*	1.60*	1.10-2.34		0.98	1.18	0.78-1.78		1.28	1.33*	1.00-1.75
Middle		1.42	1.29	0.90-1.86		0.97	1.25	0.85-1.82		1.20	1.24	0.96-1.60
Fourth		1.24	1.33	0.90-1.94		1.27	1.33	0.93-1.91		1.25	1.29*	1.00-1.67
Highest		1.40	1.27	0.85-1.90		1.28	1.32	0.90-1.95		1.37*	1.30	0.99-1.72
**Lack of companionship**^a^												
Never**^rc^**		1.00	1.00	.		1.00	1.00	.		1.00	1.00	.
Rarely		1.06	1.05	0.84-1.31		0.96	0.97	0.78-1.22		1.06	1.05	0.89-1.23
Sometimes		0.97	1.20	0.93-1.54		0.61**	0.61**	0.44-0.86		0.74*	0.81	0.65-1.01
Often^b^		0.66*	0.80	0.56-1.14		0.65*	0.77	0.53-1.14		0.67**	0.78	0.61-1.01
**Frequency of contact with kin**^c^												
At least weekly/Monthly**^rc^**		1.00	1.00	.		1.00	1.00	.		1.00	1.00	.
Twice a year		1.02	0.99	0.79-1.22		0.94	0.91	0.65-1.28		0.94	0.93	0.77-1.13
Once a year		0.96	0.97	0.74-1.28		1.72**	1.40	0.97-2.01		1.27*	1.17	0.96-1.44
Very rarely		0.83	0.84	0.67-1.04		1.21	1.11	0.84-1.45		0.97	0.94	0.80-1.10
**Functional status**												
Limited**^rc^**			1.00	.			1.00	.			1.00	.
Not limited			2.38***	1.67-3.38			3.57***	2.47-5.15			2.95***	2.26-3.85
**Observations**			**322**				**313**				**635**	
Link test hat (p-value)			0.000				0.000				0.000	
Link test hatsq ((p--value)			0.666				0.193				0.151	
Model p-value			0.013				0.000				0.000	
Log likelihood			-268.95				-230.31				-505.10	

**^rc^**Reference category, ***aPR*** Adjusted Prevalence Ratio, ***CI*** Confidence Interval, Significant (*p* < 0.05), *p < 0.05 (moderate), **p < 0.01 (strong), ***p < 0.0001 (very strong), survey weights were applied (svy) ^a^Lack of companionship in the past four (4) months. ^b^3+ times a week. ^c^Contact through the phone, electronic ways, post, and physical meetings.

In Model II, where males and females were analysed together, females compared with males (aPR = 0.81) and Kampala district compared with Wakiso (aPR = 0.83) consistently remained significantly associated with a lower likelihood of reporting good SRH. Similarly, older adults who reported a lack of companionship more than rarely had a lower likelihood of reporting good SRH than those who never lacked companionship. The adjusted prevalence rate decreased by approximately 30%. Other factors that showed significant evidence of a higher likelihood of reporting good SRH included satisfaction with accommodation (aPR = 1.66), working (aPR = 1.33), lack of house ownership by the household (aPR = 1.21), reporting the highest wealth quintile (aPR = 1.37), and contact with kin once a year (aPR = 1.27). In the female-stratified model, those who reported being satisfied with accommodation (aPR = 1.83) and those who reported having contact with kin once a year (aPR = 1.72) were more likely to report good SRH. Regarding lack of companionship, females who reported lack of companionship sometimes (aPR = 0.61) or often (aPR = 0.65) had a decreased likelihood of reporting good SRH. In the male-stratified model, a positive increase in the likelihood of reporting good SRH was observed for those satisfied with accommodation (aPR = 1.58), secondary-level education (aPR = 1.26), working (aPR = 1.55), and those who reported being in the second wealth quintile (aPR = 1.62). Males in an alternative family structure, affiliated with the Protestant Church, and who often lacked companionship had a lower likelihood of reporting good SRH. The adjusted prevalence rates reduced by 28%, 20%, and 34%, respectively.

In the full model, which combined males and females, Table 4 shows that older adults residing in Kampala consistently had a lower likelihood of reporting good SRH. The adjusted prevalence rates were reduced by 17% (aPR = 0.83; 95% CI = 0.72–0.97). The percentage increase for other factors which were positively associated with good SRH are summarised as 57% for older adults satisfied with accommodation compared to not (aPR = 1.57; 95% CI = 1.27–1.93); 20% for those who were working compared with those who were not working (aPR = 1.20; 95% CI = 1.00–1.44); 18% for those who reported not living in a house owned by the household compared to those living in a house owned by the household (aPR = 1.18; 95% CI = 1.00–1.39); 33% for those who were in the second wealth quintile compared to lowest (aPR = 1.33; 95% CI = 1.00–1.75); 29% for older adults with the fourth wealth quintile compared to lowest (aPR = 1.29; 95% CI = 1.00–1.67); and 195% for older adults whose functional status was not limited compared to limited (aPR = 2.95; 95% CI = 2.26–3.85). In the female-stratified model, Kampala, compared to Wakiso district, and older adults who reported lacking companionship sometimes, compared with never, had a lower likelihood of reporting good SRH. The adjusted prevalence rates decreased by 23% and 39%, respectively (aPR = 0.77; 95% CI = 0.60–0.98 vs aPR = 0.61; 95% CI = 0.44–0.86). Factors associated with a higher likelihood included satisfaction with accommodation (aPR = 1.53) and having no functional limitations (aPR = 3.57). In the male-stratified model, older adults in the alternative family structure compared to the nuclear family structure, and Protestants compared to Catholics had a lower likelihood of reporting good SRH by 26% and 22%, respectively (aPR = 0.74; 95% CI = 0.57–0.94 vs aPR = 0.78; 95% CI = 0.63–0.96). Furthermore, there was a higher likelihood of reporting good SRH for older males who reported that they were satisfied with their accommodation (aPR = 1.59; 95% CI = 1.25–2.03), working (aPR = 1.41; 95% CI = 1.03–1.93), those in the second wealth quintile compared to the lowest (aPR = 1.60; 95% CI = 1.10–2.34), and those without limited functional status compared to limited (aPR 2.38; 95% CI = 1.67–3.38). Although the combined model (for both males and females) uses the complete analytical sample (*n* = 651) to yield robust estimates at the population level, findings from the sex-stratified models present detailed statistical insights into sex-stratified patterns. However, sex-stratification reduces stratum-specific cell sizes, and the resulting estimates may be unstable due to limited statistical power. Therefore, sex-stratified estimates should be interpreted with caution as suggestive, exploratory associations rather than definitive findings.

The diagnostic test results for the respective full models show that the observed data mirror the expected data for males (hat: *p* = 0.000; hatsq: *p* = 0.666), females (hat: *p* = 0.000; hatsq: *p* = 0.193), and for the combined model (hat: *p* = 0.000; hatsq: *p* = 0.151).

## Discussion

This study addressed three objectives. First, we estimated the prevalence of good self-reported health. Second, we explored the factors associated with good self-reported health among older adults aged 50 to 64 in a metropolitan setting. Third, we assessed sex-stratified patterns. While analysis of patterns within the sex-stratified models permits the correlates to vary across demographic, socioeconomic and health-related factors, the stratum-level empirical variations should be interpreted with caution as suggestive and exploratory rather than definitive, because the sex-stratified estimates may be unstable due to limited statistical power.

More than half (58%) of people aged 50–64 reported good SRH (64% males, 52% females). This finding is similar to that of previous studies conducted in Uganda and elsewhere [10,83]. Two arguments can explain this result. First, older women may report poorer self-reported health because they could be confronted with greater social, emotional, and economic vulnerability attributed to marital separation, divorce, or widowhood [84]. Second, it could be due to the perpetual burden of caring for grandchildren amid limited resources [85].

Our analysis revealed a strong and significant relationship between self-reported health and functional status. These results are consistent with those of Tetteh et al., who found evidence associating higher levels of functional limitations with poor SRH in Ghana, indicating that adults (≥50 years) with better functional status were more likely to report good health [83]. Our study findings may be explained by the following arguments: First, older adults who are not functionally limited may be able to engage in essential and instrumental activities of daily living, economic and social roles autonomously. For instance, they may be able to participate socially in associations or organisations, which helps them avoid the mental shift towards depression. A study by Choi (2021) revealed that social participation reduced depression among older adults aged 60 and older [86]. Moreover, social participation enhances older adults’ mental and physical health [87]. Second, older adults in the metropolitan area may have better access to healthcare services, information, and even treatment alternatives, which may enable the effective management of older adults’ health concerns and maintain good health. For instance, existing literature shows that urban residents have higher health information and access to healthcare services [88,89]. Functional status among older adults is a key factor associated with SRH, demonstrating no significant variation in SRH across sex [90,91]. Functional status reflects the overall physical abilities that influence health outcomes among older adults [92,93]. Thus, our study results suggest consolidation and further expansion of older adults’ access to rehabilitation services, community-based support, and assistive devices to mitigate the adverse health challenges of functional limitations and maintain older adults’ good health in the metropolitan area. Functional status and self-reported health are two closely related health dimensions included in the fully adjusted model. Thus, functional status’s adjusted prevalence ratios (aPRs) should be interpreted with caution, as conditional relationships revealing physical ability within a wider multidimensional health framework, rather than an explicitly separate correlate of self-reported health.

There was a positive relationship between satisfaction with accommodation and SRH among older adults. This finding concurs with those of Gordon et al., who argued that in the United States of America (State of Jersey), good health was associated with accommodation satisfaction [94]. Two perspectives may help explain this finding. First, internal housing conditions, including the number of rooms, amount of light received in the house, noise, leaky roof, crime in the neighbourhood, damp walls, and deterioration in the floor and window edges [95], can be associated with the health of older adults. For instance, poor housing conditions may trigger chronic conditions and even worsen existing complications such as asthma, resulting in poor health. A cohort investigation by Santos et al. (2025) established that older adults (60 years and older) living in poor internal housing conditions such as insufficient daylight, pollution, leaks, dampness, and rot were associated with poor quality of life, loneliness, and reduced self-reported healthy ageing [96], which could be linked to the onset of chronic lung diseases and asthma exacerbation. On the other hand, good housing conditions can prevent diseases and increase the quality of life of individuals, including older adults [97]. The second perspective concerns depressive symptoms and stress levels among older adults, which decrease with increasing satisfaction with the residential environment [98]. Satisfaction with accommodation is important in influencing the health of older adults, because it reduces stress and anxiety by providing housing that is secure and safe [99,100], which may be attributed to reduced financial strain. Therefore, this finding informs GKMA policy to focus on improving housing conditions for older adults, for instance, through improved sanitation and affordable housing, to enhance their health.

The district of residence was associated with self-reported health among older females and among all older adults. Females in Kampala district were less likely to report good self-reported health than those in Wakiso district. While Mukono and Kampala districts have slums, more than half of Kampala residents, including older adults, live in unplanned settlements, such as slums [101,102]. Slums are generally characterised by poor living conditions [103]. One possible explanation is that both older males and females are affected by the poor living conditions in the slums of Kampala district. Thus, a decrease in the likelihood of reporting good self-reported health among older females could be partly explained by poor health conditions [104,105], cost, and the accessibility of healthcare facilities [101]. This study suggests enhancements to door-to-door health screening and improvements in living conditions, such as the development of age-friendly support infrastructure.

There was a negative association between alternative family structure and good self-reported health among older males. Older men in the alternative family structure group had a lower likelihood of reporting good self-reported health. In Ugandan society, just as elsewhere, as explained by Pezzin and Torres, changes in the social and economic settings of the family structure raise uncertainty, which eventually affects individuals and families [106,107]. For instance, according to Wu, kinless older adults were found to be associated with a higher care deficit [108], although they can still reach out to friends for health support [109–111], which can boost their health. Therefore, sex differences may be explained by the argument that older males in an alternative family structure may not benefit from a stable, two-layered support system comprising their spouses and children, who may provide emotional and health support. In contrast, older females may benefit from emotional and health support provided by children, social networks, and the community [112–114]. However, we are aware that merging categories of alternative family structure limits precise understanding of the underlying mechanism.

Our study showed that females and all older adults who reported sometimes and often feeling a lack of companionship had a lower likelihood of reporting good SRH than their counterparts who never lacked companionship. This result is similar to that of a previous investigation conducted by the University of Michigan among people aged 50–80 years, disproportionately affecting women [115]. A possible explanation for our finding could be linked to a study by Stephanie and Lawton among older adults (50–70 years), who showed that companionship is among the most important factors in lowering stress among older adults, including females [116–118]. However, literature shows that older women are more likely than men to rely on close friendships and family relations for emotional, relational, and coping support [119,120]. Lack of companionship among older adults has been associated with poor mental and physical health [115]. Unlike older females, older males primarily depend on their spouses for emotional support, and the absence of it can negatively affect their health outcomes [114]. Therefore, companionship among older women and all adults should be enhanced through community-level engagement, such as faith-based outreach teams, age-friendly infrastructure, and strengthened peer support groups.

A positive relationship was observed between working and reporting good SRH among males and, in general, among all older adults. Previous studies have highlighted the importance of work. They noted that not working was associated with a higher likelihood of poor SRH [83,121]. They argue that older males are less likely than younger males to consider their work or job stressful [122]. In addition, Li et al. (2021) argued that working among older adults boosts their mental and physical abilities [123]. Three arguments can explain this finding. The first is economic and financial stability, which helps them gain better access to medical care and preventive check-ups and buy necessary medication [31,124]. Second, psychological and social benefits open access to wider social networks and resources, thereby enhancing health [125,126]. Third, employment promotes physical and cognitive well-being, which is pivotal for health [127,128].

The lower likelihood of good SRH among Protestants than Catholics may reflect differences in religious practices rather than in theological doctrine. While previous studies are inconclusive on this, some argue that Catholics report a significantly increased likelihood of poor SRH, while others report poor SRH for both Catholics and Protestants [129,130]. There is scarce literature to explain this result, although it could be associated with religious systems, practices and beliefs [114,131–133]. However, our findings should be interpreted as preliminary and best treated as hypotheses that need further research.

The higher levels of reporting good SRH among older adults who were not living in a household-owned house (renting/rent-free) compared to those living in a household-owned house is surprising. This finding contradicts the overwhelming consensus that renting is associated with a lower likelihood of reporting good SRH [134]. Limited studies explain this result in the GKMA. However, this could be attributed to their proximity to services, including healthcare services and the possibility that they may not necessarily be the renters, but rather be cared for by their adult children or relatives who provide support. Literature shows that older people who relocate closer to their adult children are more likely to move to larger metropolitan areas with better ageing-friendly services [135], and are more likely to report good physical and mental health [136]. Further investigations should be undertaken to understand the interplay between good health and not living in a household-owned house among older adults in metropolitan areas.

According to the wealth-health gradient phenomenon, there is overwhelming consensus in both developing and developed countries, where individuals in higher wealth quintiles are significantly more likely to report good self-reported health [137,138]. Although there is mixed evidence among males, the findings partly support this hypothesis. This can be attributed to three arguments. First, access to resources enables them to obtain high-quality healthcare and a better living environment without financial stress. Second, financial security among the rich reduces the stress of covering medical expenses and anxiety about outliving savings, which are often affected by market fluctuations. Third, accumulating wealth from a young age is often associated with better education and healthier behaviours, which in turn contribute to better health outcomes with ageing.

Our study revealed that age and marital status were not significantly associated with good self-reported health at the multivariable level of analysis. Education level and frequency of contact with kin lost significant association with self-reported health status at the multivariable level of analysis.

Age, marital/union status, family structure, house ownership, and religious affiliation were retained, despite not being statistically significant at the bivariate level. This was done to maintain their theoretical relevance. The significance of family structure, house ownership, and religious affiliation in the final model reveals predictors that entail sex-specific and general pathways, demonstrating the need to translate the retained and revealed significant predictors within a wider explanatory paradigm. The identified predictors indicate patterns warranting further study.

## Strengths and limitations of the study

The strengths of the study included the following: First, this study provides a yardstick for future investigations. There are limited investigations conducted in the GKMA in Uganda that aim to explore the prevalence and factors associated with self-reported health status among older adults, alongside sex-stratified analysis. The study outcomes complement the available literature on self-reported health status among older adults, including sex-stratified analyses, in a metropolitan setting. Second, the study uses a cross-sectional, representative sample of urban residents in the Greater Kampala Metropolitan Area.

The limitations of the study include the following: First, the study used cross-sectional survey data, which do not permit cause-and-effect relationships. It is difficult to establish causality in these assessments [139]. Second, the study depended on self-reported health estimates, which may be subject to bias. Third, there may have been unobserved confounders, such as cultural norms and expectations and access to healthcare services, that could have influenced the associations. Fourth, the sex-stratified results should be interpreted as suggestive and exploratory rather than definitive, since subgroup estimates may be unstable due to reduced power. Lastly, this study used data from only older adults aged 50–64 years, excluding those aged 65 and older, and thus recommends that future studies focus on people aged 65 and older in the GKMA. A qualitative approach could also explain lived experiences and context, especially regarding a lack of companionship among older males and family structures among all older adults in the GKMA, and explore the relationship between household-house ownership and self-reported health status among older adults in the GKMA.

## Conclusions

This investigation contributes to a strong insight into good self-reported health status among older adults in the GKMA, alongside sex stratification. In contrast, accommodation satisfaction, functional status, district of residence, family structure, lack of companionship, working status, household ownership, religious affiliation, and wealth quintile were associated with self-reported health status among older adults in the GKMA. The Government of Uganda, including the Ministry of Gender, Labour and Social Development, and other stakeholders, needs to appreciate the significant variations in approaches to enhancing the well-being of older adults. For instance, community-centred social care systems that emphasise companionship, improved housing conditions, enhanced age-friendly infrastructure, psychological support, income generation, and involvement, particularly for older men and women (50–64), and improved primary healthcare systems in the GKMA. Sex-stratified estimates should be interpreted with caution as suggestive and exploratory rather than definitive, given the potential for unstable estimates due to limited statistical power.

## Supplementary Material

STROBE_checklist_cross_sectional_Clean.docx

## Data Availability

The dataset supporting the conclusions of this study is available upon reasonable request from the corresponding author.
